# ROptimus: a parallel general-purpose adaptive optimization engine

**DOI:** 10.1093/bioinformatics/btad292

**Published:** 2023-05-04

**Authors:** Nicholas A G Johnson, Liezel Tamon, Xin Liu, Aleksandr B Sahakyan

**Affiliations:** Radcliffe Department of Medicine, MRC WIMM Centre for Computational Biology, MRC Weatherall Institute of Molecular Medicine, University of Oxford, Oxford OX3 9DS, United Kingdom; Radcliffe Department of Medicine, MRC WIMM Centre for Computational Biology, MRC Weatherall Institute of Molecular Medicine, University of Oxford, Oxford OX3 9DS, United Kingdom; Radcliffe Department of Medicine, MRC WIMM Centre for Computational Biology, MRC Weatherall Institute of Molecular Medicine, University of Oxford, Oxford OX3 9DS, United Kingdom; Radcliffe Department of Medicine, MRC WIMM Centre for Computational Biology, MRC Weatherall Institute of Molecular Medicine, University of Oxford, Oxford OX3 9DS, United Kingdom

## Abstract

**Motivation:**

Various computational biology calculations require a probabilistic optimization protocol to determine the parameters that capture the system at a desired state in the configurational space. Many existing methods excel at certain scenarios, but fail in others due, in part, to an inefficient exploration of the parameter space and easy trapping into local minima. Here, we developed a general-purpose optimization engine in R that can be plugged to any, simple or complex, modelling initiative through a few lucid interfacing functions, to perform a seamless optimization with rigorous parameter sampling.

**Results:**

ROptimus features simulated annealing and replica exchange implementations equipped with adaptive thermoregulation to drive Monte Carlo optimization process in a flexible manner, through constrained acceptance frequency but unconstrained adaptive pseudo temperature regimens. We exemplify the applicability of our R optimizer to a diverse set of problems spanning data analyses and computational biology tasks.

**Availability and implementation:**

ROptimus is written and implemented in R, and is freely available from CRAN (http://cran.r-project.org/web/packages/ROptimus/index.html) and GitHub (http://github.com/SahakyanLab/ROptimus).

## Graphical Abstract

**Graphical Abstract:**
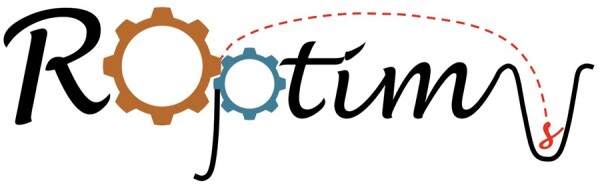

## 1 Introduction

**Figure 1.**
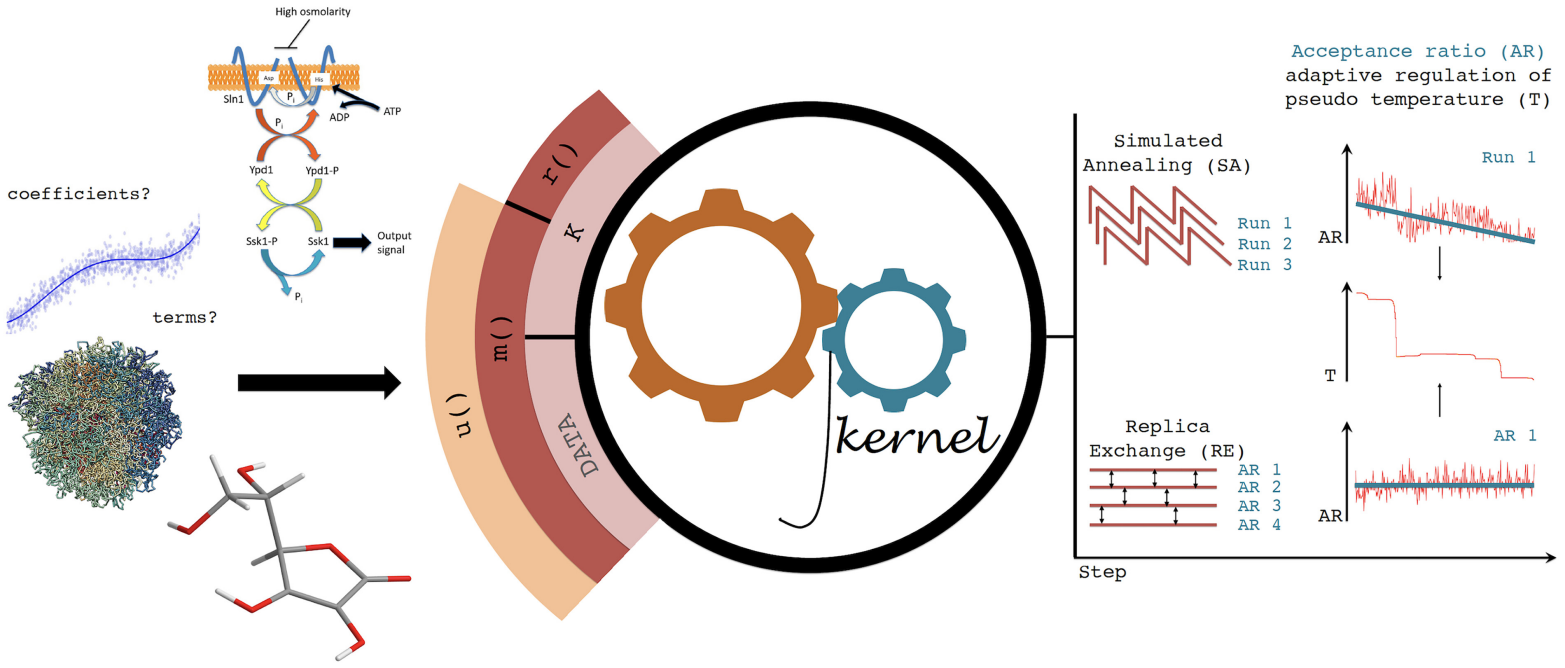
Schematic illustration of capabilities and several computational biology optimization use cases, where ROptimus engine is showcased in the Supplementary data. A modelling initiative is expressed via three fully user-defined functions operating on user inputs, K and the optional DATA object, and to interface with ROptimus SA or RE procedures. The reduced plots at the right edge represent the abrupt adaptive changes in pseudo temperature, automatically performed in ROptimus as necessitated by the evolving system to keep up with the desired gradual decrease in acceptance ratio or AR (downward trendline) shown for the last acceptance ratio annealing cycle of a representative SA run, and the desired fixed acceptance ratios (horizontal trendline) for the representative RE run. For the exchange to happen, RE requires parallel optimization (i.e. multiple cores) using different user-defined AR values while SA optionally can perform multiple runs in parallel using different seeds (internally defined based on a single input seed) when multiple cores are provided.

For a complex model, where the unknown parameters cannot be determined by conventional linear or non-linear fitting techniques (Bethea et al. 1985; Freedman 2009), optimization methods based on biased random sampling of the parameter space are the methods of choice (Cowles and Carlin 1996). In such cases, the quality of the solution found, following some optimization protocol, depends on that protocol’s ability to effectively explore the parameter space (Gilks 1996) and be drawn to more favourable areas therein. Monte Carlo-based algorithms are suitable for such biased sampling of the parameter space, generalizable for many complex optimization problems, where the specialized algorithms are impractical to use. To meet the need of a transferrable Monte Carlo-based optimization engine, where the flexibility of the usage is prioritized, and no major prior or runtime intervention of the core optimization regimen is needed by a user, here we present ROptimus—a universal and flexible optimization engine in R (R Core Team 2015) that can interface with a wide variety of modelling initiatives through a small R scripting interface. Because the appropriate temperature scale or annealing scheme can drastically vary across models or states of a system in a model, ROptimus was built to not require any situational tuning of the pseudo-temperature scaling while defining the acceptance probabilities, even if the same system is trapped into a significant local minimum. This is achieved by driving the optimization process through the annealing or replica exchange of the constrained factual acceptance ratio that automatically adapts the unconstrained pseudo temperature of the system (Fig. 1). Overall, this wide-ranging applicability due to a flexible interface, and extensive and transferrable optimization process via acceptance ratio-based automatic temperature control and an all-purpose replica exchange option differentiate ROptimus from other related multi-purpose and specialized R packages, e.g. NMOF (Gilli et al. 2019), GenSA (Xiang et al. 2013), optimization (Husmann et al. 2017) and stats (R Core Team 2015), enumerated in a comprehensive and diverse list of general-purpose and specific optimization solvers (CRAN Task View: Optimization and Mathematical Programming Version: 12 April 2022, https://cran.r-project.org/web/views/Optimization.html) (Schwendinger and Borchers 2022) and identified from the survey of the literature. Below is the brief description of the package and its major distinctions, all further detailed in the Supplementary Information bringing five tutorials to showcase the usage of the engine in diverse scenarios. It is possible that at select resource allocation, ROptimus may or may not perform better than tailored packages for specific tasks, but these tutorials demonstrate its strength as a flexible optimization engine for any model system with rigorous parameter search process regardless of the configuration of the problem.

## 2 Software description

If using the acceptance ratio simulated annealing (Kirkpatrick 1984) (AR-SA) mode of ROptimus, in each annealing cycle, ROptimus constructs a linear target AR regimen across each step. This is done based on an initial target AR, a final AR, and the number of iterations in each cycle for a given optimization run (all of which can be modified from defaults as advanced inputs). Once the optimization process begins, ROptimus calculates an observed AR at the end of each window of a fixed number of steps by calculating the fraction of the accepted moves from all the past trials in the current window at a given initial pseudo temperature. Thereafter, ROptimus compares the observed AR with the pre-defined target AR based on the annealing schedule, and determines whether and how to alter the system pseudo temperature (adaptive thermoregulation) to align the observed AR with the target ratio at the end of the following statistics window (Fig. 1). Thus, by employing AR-SA and adaptive thermoregulation, ROptimus can methodically explore the parameter space even when no smooth relationship exists between the parameters and system pseudo energy, avoiding a danger to fall into traps with no crossing with conventional methods under constrained temperature range (Ingber 1993).

ROptimus additionally supports acceptance ratio replica exchange (AR-RE) as an optimization mode, provided that the user has access to multiple processors (preferably eight or more). This additional mode was adapted from the parallel tempering/replica exchange methodologies (Swendsen and Wang 1986; Sugita and Okamoto 1999). ROptimus modifies the Monte Carlo flavour of the replica exchange approach to apply to any arbitrary optimization problem with two primary modifications: the use of varying replicas of fixed AR optimizations, and necessitated unconditional exchange after two candidate configurations in those replicas are selected for an exchange (Ballard and Jarzynski 2009). These changes are only acceptable if relaxing the equilibrium sampling criteria, whereby the parameter space can be more extensively explored for the sole reason of finding an optimal solution. This approach produces good results, as illustrated in the tutorials, and is a viable alternative to the AR-SA mode in ROptimus.

## 3 Conclusion

In cases where (i) we do not deal with energies and temperatures that emulate real physical systems; (ii) we are only interested in final optimized configuration of the system, and do not need to characterize the statistical ensemble of reachable states/solutions, we can then cross barriers in the solution energy landscape by annealing or replica exchanging based on a metric (AR) that is more transferable to different volatile system configurations. We demonstrate the usability and flexibility of ROptimus in five example tutorials (Fig. 1 and Supplementary Information): (i) optimizing coefficients to a known equation (Tutorial 1 in Supplementary Information), (ii) optimizing an equation itself by attempting various configurations of its functional form (Tutorial 2 in Supplementary Information), (iii) optimizing a geometry of a molecule while interfacing with an external quantum chemical program (Tutorial 3 in Supplementary Information), (iv) optimizing parameters to coupled ODEs from systems biology (Tutorial 4 in Supplementary Information), and (v) optimizing a highly specific constrained shuffling problem from 3D genome biology (Tutorial 5 in Supplementary Information). All these are showcased in Supplementary Information, with the full walkthrough of both the AR-SA and AR-RE variants. We successfully used this program in diverse research objectives, including in a recently published tuning of a model for the inference of iDNA stabilities (Cheng et al. 2021), and we hope it will be equally useful and accessible for other researchers.

## Supplementary Material

btad292_Supplementary_Data

## Data Availability

The data underlying this article are available in Supplementary data. ROptimus is freely available from CRAN (http://cran.rproject.org/web/packages/ROptimus/index.html) and GitHub (http://github.com/SahakyanLab/ROptimus) repositories.
